# Isolation and Characterization of *Brevibacillus parabrevis* S09T2, a Novel Ochratoxin A-Degrading Strain with Application Potential

**DOI:** 10.3390/foods15020295

**Published:** 2026-01-14

**Authors:** Jinqi Xiao, Qingping Wu, Junhui Wu, Xin Wang, Shixuan Huang, Xiaojuan Yang, Xianhu Wei, Youxiong Zhang, Xiuying Kou, Yuwei Wu, Ling Chen

**Affiliations:** 1 College of Food Science, South China Agricultural University, Guangzhou 510642, China; x1659671893@163.com; 2 State Key Laboratory of Applied Microbiology Southern China, Guangdong Provincial Key Laboratory of Microbial Safety and Health, National Health Commission Science and Technology Innovation Platform for Nutrition and Safety of Microbial Food, Key Laboratory of Big Data Technologies for Food Microbiological Safety, State Administration for Market Regulation, Institute of Microbiology, Guangdong Academy of Sciences, Guangzhou 510070, China; wuqp203@163.com (Q.W.); wujunhui@gdim.cn (J.W.); wangxin@gdim.cn (X.W.); huangshixuan@gdim.cn (S.H.); yangxiaojuan@gdim.cn (X.Y.); weixh@gdim.cn (X.W.); zhangyouxiong@gdim.cn (Y.Z.); 3 Food and Drug Laboratory, Guangdong Detection Center of Microbiology, Guangzhou 510070, China; 4 Infinitus (China) Company Ltd., Guangzhou 510623, China; xiuying.kou@infinitus-int.com; 5 Guangdong Huankai Biological SCI&TECH Co., Ltd., Zhaoqing 526238, China

**Keywords:** ochratoxin A, *Brevibacillus parabrevis*, mycotoxin detoxification, biological control

## Abstract

Ochratoxin A (OTA), a fungal secondary metabolite, is frequently detected in grains, herbal products, and other agricultural commodities, posing potential food safety risks. Among existing detoxification strategies, biological degradation is considered both specific and environmentally sustainable. In this study, a novel OTA-degrading bacterium, *Brevibacillus parabrevis* S09T2, was isolated from soil using OTA as the sole carbon source. The strain exhibited no hemolytic activity and carried no virulence or antibiotic resistance genes, indicating a favorable safety profile. S09T2 efficiently degraded OTA, removing over 93% of 5–8 μg/mL OTA within 24 h at 37 °C, and almost completely degrading OTA concentrations up to 10 μg/mL within 72 h. UPLC-HRMS analysis identified ochratoxin α (OTα) and phenylalanine as the only degradation products, confirming detoxification via amide bond hydrolysis. The intracellular enzyme responsible for this reaction displayed notable thermostability, achieving near-complete degradation of 1 μg/mL OTA at 50 °C within 6 h. Moreover, the cell lysate significantly reduced OTA levels in *Plumeria rubra* extract, a widely consumed functional food, demonstrating applicability in complex food matrices. Collectively, these findings highlight S09T2 as a promising candidate for OTA detoxification and support its potential use in food and feed safety applications.

## 1. Introduction

Ochratoxins represent a group of toxic secondary metabolites mainly synthesized by specific fungi belonging to the genera *Aspergillus* and *Penicillium* [1]. More than twenty structural analogs have been identified within this toxin family, among which ochratoxin A (OTA), ochratoxin B (OTB), and ochratoxin C (OTC) are the most intensively investigated [2]. Among these, OTA is considered the most hazardous, as it can damage the kidney and liver, suppress immune function, and induce carcinogenic effects. Consequently, the International Agency for Research on Cancer (IARC) has categorized OTA as a Group 2B agent, suggesting possible carcinogenicity to humans [3,4]. Because of its remarkable chemical stability, OTA can persist throughout food processing and storage. It is widely detected in cereals, coffee, tea, dried fruits, grapes and their derived products, as well as in functional herbal preparations and animal-derived commodities [2,5,6,7]. For instance, Banahene et al. [8] analyzed 520 cocoa bean samples from cocoa-growing regions in Ghana and reported a 21.7% OTA positivity rate, with levels ranging from 0.01 to 12.36 μg/kg. In another investigation, all 19 commercial dry red wines originating from Poland, Spain, and France were contaminated with OTA, with concentrations exceeding 6.176 μg/L [9]. Additionally, a recent systematic review summarizing OTA contamination in wheat and flour worldwide between 2000 and 2023 revealed pronounced regional differences, with Asia exhibiting the highest contamination rate at 44% [10]. To mitigate potential risks to consumer health, the European Food Safety Authority (EFSA) has established strict maximum OTA limits for major food categories, including 5 μg/kg in cereals, 3 μg/kg in cereal-derived products, 5 μg/kg in roasted, ground, and instant coffee, 3 μg/kg in cocoa powder, and 2 μg/kg in wine [11].

Numerous studies have shown that fungi such as *Aspergillus niger*, *Aspergillus ochraceus*, and *Aspergillus carbonarius* are capable of producing OTA at temperatures between 20 °C and 37 °C [12,13,14]. With global climate change driving increases in average temperatures, prolonged warm seasons, and greater humidity fluctuations. Such environmental shifts may facilitate the spread of OTA-producing fungi into colder regions previously unsuitable for their growth, elevating contamination risks in traditionally low-risk areas [15,16]. These altered climatic conditions may also enable OTA-producing fungi to establish new ecological niches or increase their incidence in regions where they are already present [7]. In addition to temperature- and humidity-related effects, rising atmospheric CO_2_ concentrations have been shown to intensify OTA-related risks. Cervini et al. [17] demonstrated that elevated CO_2_ markedly increased the colonization rate of *A. carbonarius* ITEM 5010 and stimulated OTA biosynthesis under simulated climate change conditions. Similarly, Llobregat et al. [18] found that *A. carbonarius* produced significantly higher OTA levels when exposed to elevated CO_2_ in combination with warmer temperatures. Together, these findings suggest that climate-driven changes in thermal regimes, moisture conditions, and atmospheric composition may exacerbate fungal colonization and OTA production, promote toxin accumulation during crop storage, and contribute to the emergence of OTA contamination in commodities where it was previously uncommon [19]. Furthermore, extreme weather events—such as heavy rainfall, hail, and storms—can physically damage fruit surfaces, increasing the likelihood of fungal infection and further aggravating OTA contamination [20,21].

Given the persistence and widespread occurrence of OTA in food, feed, and herbal materials, its contamination poses a significant challenge to food safety and public health. Therefore, the search for efficient and safe detoxification methods has become an urgent priority. Existing detoxification strategies for OTA include physical, chemical, and biological approaches. Among these, biological approaches offer greater specificity, safety, and environmental friendliness, making them the most attractive option [22]. Biological removal of OTA generally occurs via two distinct mechanisms: adsorption and enzymatic degradation. The adsorption of OTA by yeasts and lactic acid bacteria is primarily attributed to interactions between OTA molecules and cell wall components, such as β-glucans, mannoproteins, and peptidoglycans [23,24,25]. However, adsorption is inherently reversible and may allow for OTA release under certain environmental conditions. In contrast, several bacteria can degrade OTA through the enzymes, resulting in irreversible detoxification. Reported microbial degradation pathways yield products including ochratoxin α (OTα), ochratoxin B (OTB), and lactone-opened OTA (OP-OTA), among which OTα is considered the most desirable due to its significantly reduced toxicity [26,27]. Therefore, hydrolyzing the amide bond of OTA to yield OTα and phenylalanine (Phe) is regarded as a particularly safe and effective detoxification pathway [28]. Enrichment and screening strategies using OTA or its structural analogs (coumarin) as the sole carbon source have been widely employed to isolate OTA-degrading microorganisms. Several strains with OTA-degrading abilities have been isolated, including *Bacillus megaterium* JSW-B1 [29], *Microbacterium esteraromaticum* ASAG1016 [30], *Bacillus velezensis* E2 [31], *Brevundimonas naejangsanensis* ML17 [32], *Brevibacillus sp.* ALJ01 and ALJ02 [33], *Cryptococcus podzolicus* Y3 [34], *Bacillus amyloliquefaciens* YL-1 [35], and *Cytobacillus oceanisediminis* CO29 [36]. Although multiple OTA-degrading microorganisms have been identified, their degradation rates vary considerably, with some strains requiring more than two days of incubation to achieve degradation efficiencies above 90% [29,31]. Such variability likely stems from differences in enzyme production levels and catalytic capacities among strains. Meanwhile, systematic evaluations of degradation performance under different OTA concentrations are still lacking, making it difficult to determine whether high OTA levels might impair the degradation ability of the strains [35,37]. More importantly, most studies focus solely on endpoint measurements, lacking kinetic analyses to clarify the dynamic transformation of OTA and the temporal relationship between its depletion and OTα accumulation, leaving uncertainty as to whether OTα is the ultimate degradation product [30]. Furthermore, safety assessments of OTA-degrading strains are generally absent, and some strains may even exhibit hemolytic activity or harbor virulence and antimicrobial resistance genes, which severely limits their potential applications in food and feed production [38].

These limitations highlight the urgent need to discover novel OTA-degrading microorganisms with both high degradation efficiency and safety for practical applications. To address this need, a novel OTA-degrading bacterial strain, designated S09T2, was isolated from soil and identified as *Brevibacillus parabrevis* through 16S rRNA gene sequence analysis. This study aimed to (1) investigate the OTA degradation efficiency of strain S09T2 and identify its degradation products using UPLC-HRMS; (2) evaluate the OTA-degrading activity of different cellular components of S09T2 and assess the stability of the active fraction under varying temperature and pH conditions; and (3) explore the application potential of S09T2 for OTA degradation in OTA-contaminated samples.

## 2. Materials and Methods

### 2.1. Chemicals and Medium

Standard solution of Otα (0.1 mg/mL) and OTA standard powders were obtained from Pribolab (Qingdao, China). The OTA stock solution (1 mg/mL) was prepared in methanol [30]. Analytical-grade methanol and acetonitrile were purchased from Tianjin Siyou Fine Chemical Co., Ltd. (Tianjin, China). Universal primers targeting the 16S rRNA gene were synthesized by HuaDa Biotechnology Co., Ltd. (Guangzhou, China). Luria–Bertani (LB) and tryptic soy agar (TSA) media were purchased from Huankai (Guangzhou, China). LB medium was prepared by dissolving 21 g of LB powder in 1 L of deionized water, while TSA medium was prepared by dissolving 40 g of TSA powder in 1 L of deionized water [39]. All other analytical-grade reagents used in this study were obtained from Guangzhou Chemical Reagent Factory (Guangzhou, China).

### 2.2. Screening and Isolating of OTA-Degrading Strain

Twelve soil samples were obtained from different locations across Shaanxi, Guangxi, and Gansu provinces in China. To isolate strains capable of degrading OTA, minimal salt medium (MSM) containing OTA as the exclusive carbon source was used for enrichment and screening [29,40].

Each soil sample (approximately 1.0 g) was suspended in 10 mL of sterile saline (0.9% NaCl) and incubated under shaking conditions (200 rpm, 37 °C) for 12 h. A 1% (*v*/*v*) aliquot of the clarified suspension was transferred into minimal salt medium (MSM) containing KH_2_PO_4_ (0.25 g/L), MgSO_4_·7H_2_O (0.25 g/L), KNO_3_ (0.5 g/L), (NH_4_)_2_SO_4_ (0.5 g/L), CaCl_2_·2H_2_O (0.005 g/L), and FeCl_3_·6H_2_O (0.003 g/L), with OTA provided as the exclusive carbon source. The inoculated cultures were maintained at 37 °C with orbital shaking (200 rpm) for 5 days. Following incubation, the residual OTA concentration in the supernatant was determined as described in Section 2.3. Samples exhibiting a significant reduction in OTA levels were serially diluted (10^−1^ to 10^−5^) with sterile water, and 100 μL aliquots from each dilution were spread onto LB plates. Following a 48 h incubation at 37 °C, distinct colonies were isolated and purified through successive streaking. The obtained strains were evaluated for their ability to degrade ochratoxin A (OTA) in LB medium supplemented with the toxin. The strain exhibiting the highest degradation efficiency was named S09T2. For long-term preservation, the purified isolate was preserved at −80 °C in 40% (*v*/*v*) glycerol.

(1) $$OTA\;residual\;rate\;(\%)=\frac{Final\;OTA\;concentration}{Initial\;OTA\;concentration}\times 100\%$$

### 2.3. Analysis of OTA and Its Degradation Product OTα

#### 2.3.1. Quantification by UPLC-MS/MS

Quantification of OTA and its degradation product OTα was performed by UPLC–MS/MS. Analyses were conducted with an LC-20 UPLC system (Shimadzu, Kyoto, Japan) coupled to a 5500+ triple quadrupole mass spectrometer (AB SCIEX, Framingham, MA, USA) operating under electrospray ionization (ESI) source. In ESI, analytes are ionized through the formation and desolvation of charged droplets under a high-voltage electric field, and the resulting ions are detected in multiple reaction monitoring (MRM) mode [41]. The optimized declustering potentials and collision energies for OTA and OTα are summarized in Supplementary Table S1. For sample treatment, 100 μL of fermentation broth was combined with 900 μL of acetonitrile containing 0.1% formic acid, briefly vortexed, and then centrifuged at 12,000× *g* for 10 min. The clarified supernatant was passed through a 0.22 μm nylon syringe filter and placed into chromatography vials.

Chromatographic analysis was carried out using an HSS T3 column (100 mm × 2.1 mm, 1.8 μm; MicroPulite, Guangzhou, China) maintained at 40 °C. The mobile phase consisted of 0.1% formic acid in ultrapure water (solvent A) and acetonitrile (solvent B). A gradient elution program was applied as follows: 10% B for the first 1 min, linearly increased to 90% B over the next 4 min, held at 90% B for 3 min, and then returned to 5% B from 8 to 10 min. The flow rate was set to 0.3 mL/min, with 2 μL of sample injected per run. The UPLC–MS/MS method for OTA and OTα was validated according to the ICH guideline [42]. The evaluated parameters included linearity, limit of quantification (LOQ), matrix effects (ME), trueness (recovery), and precision. Linearity was assessed using matrix-matched calibration curves prepared at seven concentration levels (1.0–200.0 μg/L), and regression coefficients (R^2^) were obtained by least-squares analysis. The limits of detection (LOD) and quantification (LOQ) were determined based on signal-to-noise ratios of 3:1 and 10:1, respectively. Trueness was evaluated through recovery experiments at three spiking levels (10.0, 100.0, and 1000.0 μg/L). Precision was expressed as the relative standard deviation of repeatability (RSDr), calculated from six replicate measurements (*n* = 6) performed within a single analytical run. Matrix effects were evaluated by comparing the slopes of calibration curves prepared in solvent and in matrix extract, and were calculated using the following equation:

(2) $$ME\;(\%)=(\frac{{Slope}_{matrix}-{Slope}_{solvent}}{{Slope}_{solvent}})\times 100\%$$

#### 2.3.2. UPLC-HRMS Analysis

The identification of OTA degradation products was carried out on an Orbitrap Exploris 120 high-resolution mass spectrometer (Thermo Fisher Scientific, Waltham, MA, USA) equipped with an electrospray ionization (ESI) source. Chromatographic conditions were identical to those described in Section 2.3.1. A full scan ranges from *m*/*z* 70 to 1000 was employed in both positive and negative electrospray ionization modes. The ESI parameters were adjusted as follows: sheath gas, 50 Arb; auxiliary gas, 10 Arb; capillary temperature, 320 °C; and spray voltages of +3.5 kV and −2.5 kV for positive and negative modes, respectively. Full MS scans data were obtained at a resolving power of 30,000, and MS/MS acquisition was performed at 15,000 resolution using stepped normalized collision energies of 30, 50, and 70 eV. Xcalibur 4.1 (Thermo Fisher Scientific, Waltham, MA, USA) was used for data processing and compound identification.

### 2.4. Phenotypic and Molecular Identification of S09T2 Strain

#### 2.4.1. Morphological Characteristics of S09T2 Strain

The S09T2 strain was cultivated on Luria–Bertani agar plates at 37 °C for 24 h. The appearance of colonies was visually inspected, and Gram staining was performed to examine cellular morphology under a microscope.

#### 2.4.2. Molecular Identification of S09T2 Strain

Universal primers 27F and 1492R, with sequences 5′-AGAGTTTGATCCTGGCTCAG-3′ and 5′-GGTTACCTTGTTACGACTT-3′, respectively, were employed to amplify the 16S rRNA gene from strain S09T2. The PCR products were purified and sequenced by HuaDa Biotechnology Co., Ltd. (Guangzhou, China). The resulting sequence was submitted to the NCBI database and analyzed using the BLAST+ 2.16.0. Homologous 16S rRNA sequences of related species were downloaded from GenBank, and phylogenetic tree analysis was performed using the neighbor-joining method with 1000 bootstrap iterations in MEGA version 11.

#### 2.4.3. Hemolytic Activity Assay of Strain S09T2

The hemolytic activity of strain S09T2 was evaluated on TSA plates containing 5% (*v*/*v*) defibrinated sheep blood [43,44]. After activation, 3 μL of the bacterial suspension was placed at three separate spots on the blood agar plate. *Staphylococcus aureus* ATCC 6538 served as a positive control for β-hemolysis. Following a 24 h incubation at 37 °C, hemolytic activity was determined by the formation of transparent zones surrounding bacterial growth. The formation of a distinct, transparent zone was interpreted as β-hemolysis (complete hemolysis), while the absence of such a zone was regarded as γ-hemolysis (non-hemolytic).

### 2.5. OTA Degradation by S09T2 Strain

#### 2.5.1. Detection and Identification of OTA Degradation Products by S09T2 Strain

The OTA degradation capacity of strain S09T2 was assessed based on a previously described method [31], with slight modifications. Briefly, we inoculated 0.99 mL fresh LB medium with an overnight culture of S09T2 and supplemented it with 10 μL OTA stock solution to yield a final concentration of 1 μg/mL. Samples were collected at 0, 12, 24, 48, and 72 h during incubation at 37 °C, and OTA concentrations were quantified by UPLC-MS/MS. Additionally, the sample collected at 72 h was subjected to UPLC-HRMS analysis to identify OTA degradation products.

#### 2.5.2. OTA Degradation Capacity of S09T2 Strain

To investigate the degradation kinetics of OTA by strain S09T2, an activated culture of strain S09T2 (1% *v*/*v*) was inoculated into 5 mL of LB medium containing 1 μg/mL OTA and incubated at 37 °C with 160 rpm. At predetermined intervals (0, 4, 8, 12, 16, 24, 32, 40, 48, 56, 64, and 72 h), the supernatants were collected and analyzed using UPLC–MS/MS to quantify the concentrations of OTA and OTα. The kinetic data were also examined to assess whether degradation products other than OTα were formed during OTA biotransformation.

Furthermore, to evaluate whether strain S09T2 can tolerate elevated OTA levels while maintaining its degradation capacity, 24 h fermentation broths of S09T2 were supplemented with different OTA concentrations (1, 2, 5, 8, and 10 μg/mL). The mixtures were incubated at 37 °C with 160 rpm, and samples were collected at 0, 24, 48, and 72 h for OTA quantification by UPLC-MS/MS.

(3) $$OTA\;degradation\;rate\;(\%)=(\frac{Initial\;OTA\;concentration-Final\;OTA\;concentration}{Initial\;OTA\;concentration})\times 100\%$$

#### 2.5.3. OTA Degradation by Different Components of S09T2 Strain

The OTA-degrading activity of different cellular components of strain S09T2 was assessed according to the methods of Peng et al. [32] and Yang et al. [45], with minor modifications. To generate different cellular fractions of strain S09T2, cultures were initiated in LB medium (1%, *v*/*v* inoculum) and incubated at 37 °C with 220 rpm for 24 h, and the supernatant was passed through a 0.22 μm membrane to obtain the sterile culture filtrate. The cell pellet was washed twice with sterile PBS and resuspended in the same buffer to yield a fresh cell suspension. One portion of the suspension was heat-sterilized (121 °C, 15 min) to prepare inactivated cells, whereas another was disrupted by ultrasonication on ice (600 W, 5 s on, 5 s off, 30 min total). The lysate was centrifuged (12,000× *g*, 10 min, 4 °C), and the resulting supernatant was filtered through a 0.22 μm membrane to yield the cell lysate. Subsequently, OTA was added to each fraction to a final concentration of 1 μg/mL, followed by incubation at 37 °C for 48 h. The residual OTA was analyzed using UPLC–MS/MS, and degradation percentages were subsequently calculated.

### 2.6. Analysis of the Degradation Characteristics of OTA by the Cell Lysate of S09T2 Strain

#### 2.6.1. Influence of Heat, Proteinase K, and EDTA on the OTA-Degrading Capacity of the Cell Lysate

To investigate the biochemical nature of the OTA-degrading components present in the S09T2 cell lysate, treatments were performed following the approaches described by Yang et al. [36] and Xu et al. [46], with minor modifications. The cell lysate was prepared as described in Section 2.5.3. To assess the impact of heat on OTA-degrading activity, the cell lysate was incubated in a boiling water bath at 100 °C for 10 min. Additionally, the cell lysate was treated with proteinase K (1 mg/mL) at 55 °C for 1 h to investigate the effect of proteolytic digestion on its degradation capability. To account for the thermal effect, a control sample was incubated at 55 °C for 1 h without proteinase K. In addition, a PBS solution containing proteinase K (1 mg/mL) was treated under the same conditions to assess whether the proteinase K alone could degrade OTA. To examine the potential metal ion dependence of the OTA-degrading activity, EDTA was added to the cell lysate at a final concentration of 10 mM. An untreated cell lysate sample served as the control.

OTA was added to each sample to reach a final concentration of 1 μg/mL. The mixtures were then incubated at 37 °C with 160 rpm for 24 h. After incubation, residual OTA concentrations were quantified by UPLC-MS/MS as described in Section 2.3, and the corresponding degradation percentages were determined.

#### 2.6.2. Thermal Stability of the Cell Lysate

To evaluate the thermal stability of the OTA-degrading activity, the S09T2 cell lysate was treated following the procedure described by Jia et al. [30], with minor modifications. The protein concentration of the lysate was quantified using a BCA Protein Assay Kit (Beyotime, Shanghai, China) and adjusted to 0.2 mg/mL. The lysate was then mixed with OTA to reach a final concentration of 1 μg/mL and incubated for 6 h at various temperatures (25, 30, 37, 40, 50, 60, 70, and 80 °C). Following incubation, residual OTA levels were analyzed by UPLC–MS/MS according to the procedure outlined in Section 2.3.

#### 2.6.3. pH Stability of the Cell Lysate

After the thermal stability experiment, the effect of pH on OTA-degrading activity was further examined following the procedure described by Peng et al. [32], with modifications. The S09T2 lysate was concentrated using a 3 kDa centrifugal filter unit (Millipore, Burlington, MA, USA) to a protein concentration of 4 mg/mL. Different pH environments were prepared by mixing 50 μL of the concentrated lysate with 950 μL of 0.1 mol/L buffer solutions, namely citric acid–phosphate (pH 3.0–5.0), sodium phosphate (pH 6.0–8.0), and glycine–NaOH (pH 9.0–10.0). The mixtures were incubated at 37 °C for 6 h to evaluate the influence of pH on OTA-degrading activity. After incubation, the residual OTA concentrations were quantified by UPLC-MS/MS.

### 2.7. Purification of OTA-Contaminated Plumeria rubra Extract Using the Cell Lysate of Strain S09T2

Herbal products are widely consumed as functional foods due to their perceived health benefits [47,48]. However, recent studies have indicated that OTA contamination also occurs in these products, posing a potential food safety concern [49]. To evaluate the applicability of the S09T2 cell lysate in real-world matrices, dried *Plumeria rubra* samples previously identified as OTA-contaminated in our laboratory were used for the detoxification assay. *Plumeria rubra* was sterilized at 121 °C for 20 min after grinding and sieving (20 mesh) and stored at room temperature. For extraction, 1 g of powder was suspended in 10 mL distilled water, heated at 80 °C for 1 h, sonicated for 30 min, and subsequently centrifuged at 3500× *g* for 10 min. The supernatant served as the extract.

A 1:1 (*v*/*v*) mixture of S09T2 cell lysate and herbal extract was incubated at 37 °C for 12 h, while PBS buffer was used in the control. Following incubation, 2 mL of the reaction mixture was evaporated under nitrogen, resuspended in 1 mL methanol containing 0.1% formic acid, sonicated for 30 min, centrifuged at 8000× g for 10 min, and filtered through a 0.22 μm PES syringe filter for OTA quantification by UPLC-MS/MS.

### 2.8. Statistical Analysis

All experiments were conducted in triplicate, and data are expressed as the mean ± standard deviation (SD). Statistical analysis was performed using GraphPad Prism 10.0 (USA). Data were assessed for normality using the Shapiro–Wilk test and for homogeneity of variance using Levene’s test. Group differences were evaluated using one-way ANOVA, followed by Tukey’s multiple-comparison test for normally distributed data. Values of *p* ≤ 0.05 were considered statistically significant and are indicated as *p* ≤ 0.05 (*) and *p* ≤ 0.01 (**).

## 3. Results

### 3.1. Isolation and Identification of OTA-Degrading S09T2 Strain

#### 3.1.1. Screening and Isolation of OTA-Degrading Bacteria from Soil

The validation parameters for OTA and OTα are summarized in Table S2. Both analytes exhibited good linearity within the range of 1–200 μg/L, with correlation coefficients (R^2^) greater than 0.999. The recoveries at three spiking levels (10.0, 100.0, and 1000.0 μg/L) were all above 92.91%. The RSDr values for OTA and OTα were 6.04% and 7.12%, respectively. In addition, the matrix effects were 8.19% for OTA and 11.97% for OTα. Since all RSDr values were below 20% and matrix effects within ±20% are considered acceptable [50,51], the method was deemed accurate and reliable for the quantification of OTA and OTα in solution. Twelve soil samples obtained from various regions across China were first enriched in sterile 0.9% NaCl solution and then introduced into MSM supplemented with 10 μg/mL OTA. After incubation at 37 °C for 5 days, OTA concentrations in the culture supernatants were quantified by UPLC-MS/MS to determine the residual OTA concentration. Among them, the culture derived from sample S09 showed almost complete OTA degradation, with a residual rate of only 0.7% (Figure S1A). This sample was thus selected for further isolation of OTA-degrading bacteria. A 100 μL aliquot of the diluted enrichment culture was spread onto LB plates. Following incubation at 37 °C for 48 h, colonies exhibiting distinct morphologies were selected and inoculated into LB medium containing OTA (1 μg/mL) to assess their individual degradation ability. One isolate, S09T2 strain, exhibited the highest degradation efficiency, with a degradation rate of 98% after 3 days of incubation at 37 °C (Figure S1B).

#### 3.1.2. Identification of S09T2 Strain

**Figure 1:**
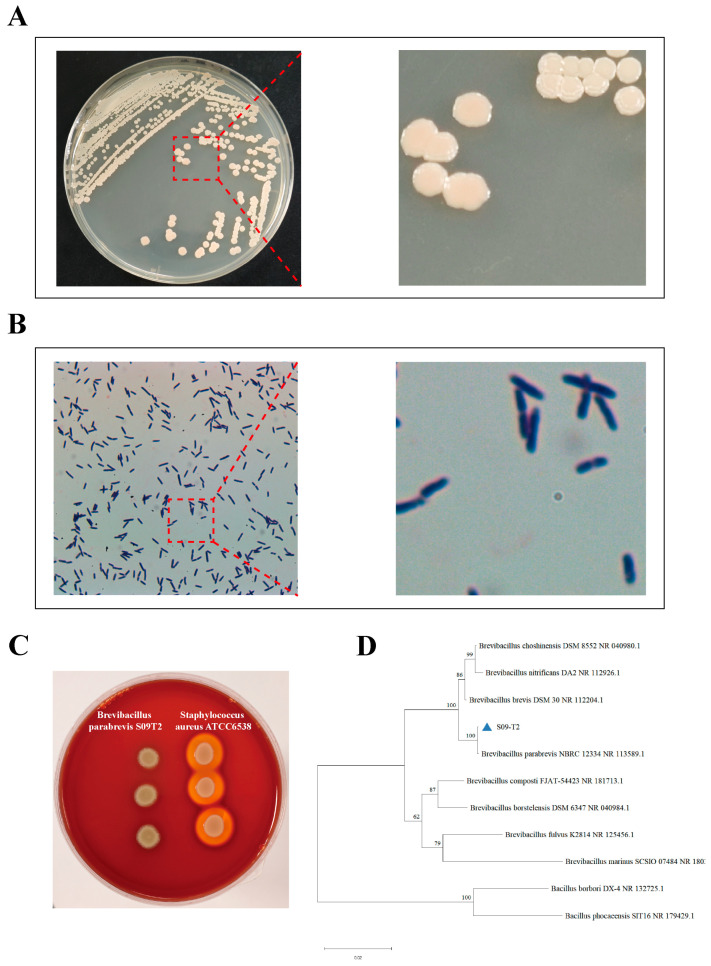
Morphological characteristics, hemolytic activity, and phylogenetic analysis of S09T2 strain. (**A**) Colony morphology of S09T2 after incubation on LB agar plates at 37 °C for 48 h. (**B**) Gram staining of S09T2 observed under a light microscope (100×, 1000×). (**C**) Hemolysis results of S09T2 and the positive control (*Staphylococcus aureus* ATCC 6538) after incubation on blood agar plates for 24 h. (**D**) Phylogenetic tree constructed based on the 16S rRNA gene sequence of S09T2 strain.

After incubation on LB plates at 37 °C for 2 days, colonies of S09T2 were round, light yellow in color, with smooth surfaces and uneven, wrinkled margins. (Figure 1A). Microscopic examination after Gram staining showed that the strain was Gram-positive and rod-shaped, typically appearing singly or in V-shaped pairs (Figure 1B). The 16S rRNA gene of S09T2 was analyzed against homologous sequences in the NCBI database. The obtained sequence was submitted to GenBank (accession no. PX056917.1), and the evolutionary relationship was visualized through a phylogenetic tree (Figure 1D). The result showed that S09T2 shared the highest similarity (>99%) with *Brevibacillus parabrevis* strain NR_113589.1, indicating that S09T2 belongs to the species *Brevibacillus parabrevis*. Notably, this strain differs from previously reported OTA-degrading bacteria.

The hemolytic activity of S09T2 is shown in Figure 1C. The left side of the plate displays colonies of S09T2, while the right side shows the β-hemolytic positive control strain *Staphylococcus aureus* ATCC 6538. No transparent zone was observed around the S09T2 colonies, indicating a negative hemolysis result. These results suggest that strain S09T2 possesses good biosafety characteristics and holds promising potential for application as an OTA-degrading bacterium.

### 3.2. OTA Degradation Characteristics of S09T2 Strain

#### 3.2.1. Identification of OTA Degradation Products by S09T2 Strain

**Figure 2:**
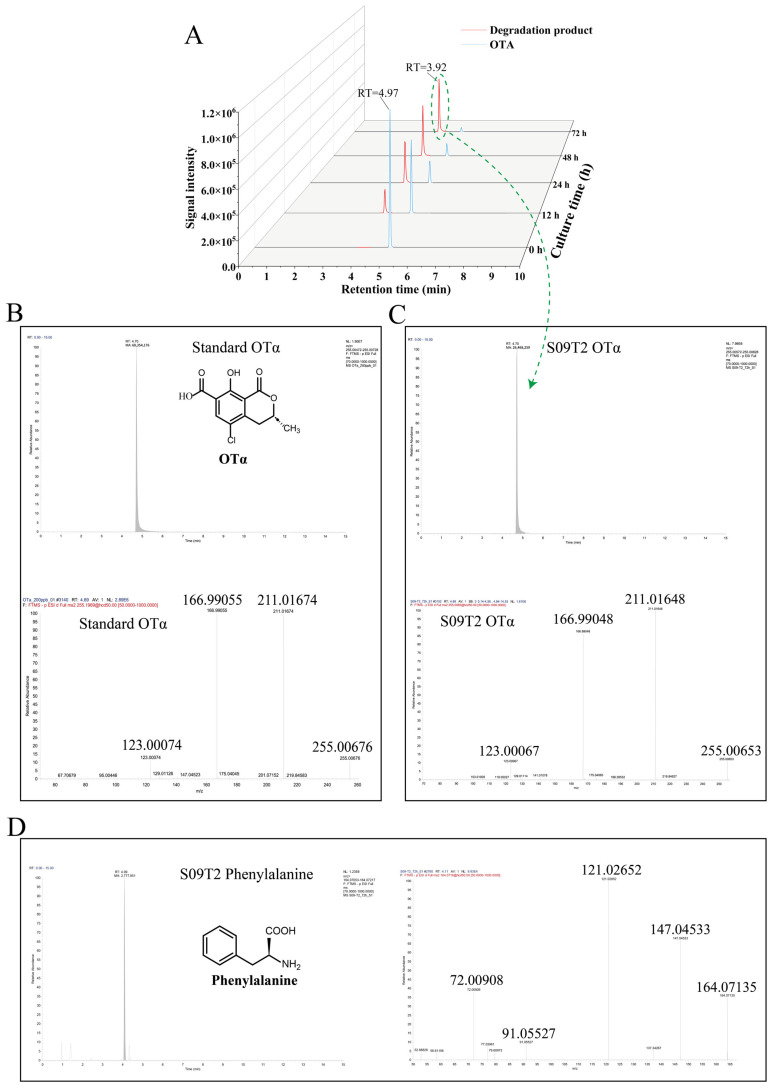
OTA degradation by strain S09T2 and identification of its degradation products. (**A**) Chromatograms showing the decrease in OTA and the appearance of degradation products over time during co-incubation with S09T2 strain, detected by UPLC–MS/MS. (**B**) Chromatogram and MS/MS fragmentation spectrum of the OTα standard detected by UPLC–HRMS in negative ion mode. (**C**) Chromatogram and MS/MS fragmentation spectrum of OTα detected in the sample after co-incubation of OTA with S09T2 strain, analyzed by UPLC–HRMS in negative ion mode. (**D**) Chromatogram and MS/MS fragmentation spectrum of the presumed degradation product phenylalanine (Phe) detected in the co-incubation sample by UPLC–HRMS in negative ion mode.

As illustrated in Figure 2A, ochratoxin A (OTA) exhibited a retention time (RT) of 4.97 min. During co-incubation with S09T2 strain, the peak area corresponding to OTA progressively declined, while a new peak appeared at RT 3.92 min and increased in intensity, indicating the generation of a degradation product. To identify this degradation products, samples collected after 72 h of incubation were analyzed using an Orbitrap Exploris 120 mass spectrometer. As illustrated in Figure 2B, the OTα standard exhibited an RT of 4.70 min under negative ion mode, with major fragment ions (*m*/*z* 255.00676, 211.01674, 166.99055, and 123.00074). After 72 h of co-incubation, a compound with the same RT (4.70 min) was detected in the S09T2-treated sample (Figure 2C), displaying a highly similar MS/MS fragmentation pattern (*m*/*z* 255.00653, 211.01648, 166.99048, and 123.00067), confirming the presence of OTα. In addition, another degradation product, phenylalanine (Phe), was detected in the same sample (Figure 2D). The observed fragment ions (*m*/*z* 164.07135, 147.04533, 121.02952, 91.05527, and 72.00908) closely matched the MS/MS fragmentation pattern reported for phenylalanine in the study by Sekimoto [52]. Taken together, these findings indicate that S09T2 degrades OTA by hydrolyzing its amide bond, producing OTα and phenylalanine as the resulting degradation products.

#### 3.2.2. Degradation Kinetics of OTA by S09T2 Strain

**Figure 3:**
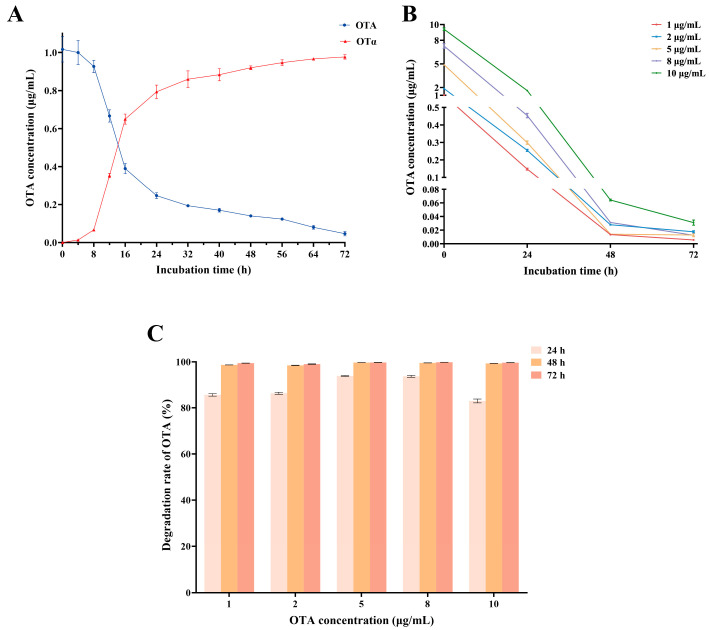
Kinetic profile of OTA degradation by strain S09T2 and its degradation efficiency at different OTA concentrations. (**A**) S09T2 strain was incubated with 1 μg/mL of OTA at 37 °C, and samples were collected at 0, 4, 8, 12, 16, 24, 32, 40, 48, 56, 64, and 72 h to determine the concentrations of OTA and its degradation product OTα. (**B**) Changes in OTA concentrations in S09T2 fermentation broth containing different initial OTA concentrations (1, 2, 5, 8, and 10 μg/mL) during incubation at 37 °C for 0, 24, 48, and 72 h. (**C**) OTA degradation rates in S09T2 fermentation broth containing different initial OTA concentrations (1, 2, 5, 8, and 10 μg/mL) during incubation at 37 °C for 0, 24, 48, and 72 h. Values are reported as the mean ± standard deviation (SD) (*n* = 3).

To determine whether OTα is the final degradation product of OTA by S09T2, the strain was co-incubated with 1 μg/mL OTA, and both OTA and OTα concentrations were determined at specific time intervals (0, 4, 8, 12, 16, 24, 32, 40, 48, 56, 64, and 72 h). The degradation kinetics were plotted accordingly. As shown in Figure 3A, OTA degradation progressed steadily over time, accompanied by a proportional increase in OTα concentration. The conversion appeared to follow a 1:1 molar ratio, with nearly all of the initial 1 μg/mL OTA transformed into an equivalent amount of OTα by 72 h. No further degradation of OTα was observed throughout the incubation period, indicating that S09T2 is unable to metabolize OTα. These findings confirm that OTα is the terminal degradation product of OTA by the S09T2 strain.

According to the OTA degradation kinetics of S09T2 strain (Figure 3A), the most rapid reduction in OTA concentration occurred within the first 24 h of incubation in LB medium at 37 °C. Based on this observation, OTA was added to the 24 h fermentation broth of S09T2 to assess its degradation efficiency. As shown in Figure 3B, after an additional 24 h of incubation at 37 °C, the degradation rates for OTA at different concentrations (1, 2, 5, 8, and 10 μg/mL) all exceeded 83%. Notably, degradation rates at 5 and 8 μg/mL exceeded 93% (Figure 3C), which were higher than those observed at other concentrations, suggesting that elevated OTA concentrations may stimulate the degradation activity of S09T2. OTA at concentrations below 5 μg/mL was almost completely degraded within 48 h, while higher concentrations required 72 h. After degradation, residual OTA levels in all samples were reduced to below 0.04 μg/mL (Figure 3B). These results indicate that strain S09T2 maintains high degradation efficiency even under elevated OTA concentrations.

#### 3.2.3. OTA Degradation Abilities of Different Components of S09T2 Strain

**Figure 4:**
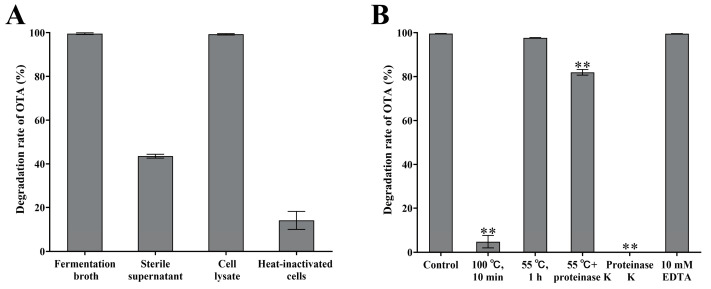
OTA degradation ability of different components of S09T2 and the effects of various treatments. (**A**) OTA degradation by the fermentation broth, sterile supernatant, cell lysate, and heat-inactivated cells of S09T2 strain. (**B**) The effects of different treatments, including heat treatment (100 °C), proteinase K (1 mg/mL), and EDTA (10 mM), on the OTA-degrading activity of the S09T2 cell lysate. The untreated cell lysate was used as the control. All reactions were conducted at 37 °C for 24 h. ** indicates *p* values < 0.01. Values are reported as the mean ± standard deviation (SD) (*n* = 3).

To investigate which components of strain S09T2 contribute to OTA degradation, the fermentation broth, sterile supernatant, cell lysate, and heat-inactivated cells were prepared and individually incubated with OTA at a final concentration of 1 μg/mL. The degradation rates of OTA were then determined. As shown in Figure 4A, the cell lysate exhibited a 99.3% OTA degradation rate, which was comparable to that of the fermentation broth and markedly higher than those observed for the sterile supernatant and heat-inactivated cells. These results suggest that the OTA-degrading activity of S09T2 mainly resides in intracellular components. The partial degradation observed in the sterile supernatant may be attributed to the release of intracellular enzymes following the natural lysis of a portion of cells during fermentation. In contrast, the weak activity of heat-inactivated cells implies that the bacterial cell surface may possess a slight OTA adsorption capacity. Taken together, these findings indicate that the OTA-degrading activity of S09T2 is primarily attributed to an intracellular component.

#### 3.2.4. Effects of Different Treatments on OTA-Degrading Activity of S09T2 Cell Lysate

As illustrated in Figure 4B, boiling the cell lysate (100 °C, 10 min) almost completely abolished its OTA-degrading activity, with the degradation rate dropping from 99.6% in the untreated control to only 4.8% (*p* < 0.01). Similarly, treatment with proteinase K (1 mg/mL, 55 °C, 1 h) significantly reduced the degradation efficiency to 81.9% (*p* < 0.01), while proteinase K alone showed no OTA-degrading activity. These results demonstrate that OTA degradation by S09T2 is mediated by an intracellular enzyme. In contrast, heating the lysate at 55 °C for 1 h without proteinase K or adding 10 mM EDTA did not significantly alter the degradation rate (*p* > 0.05). Collectively, these data indicate that the OTA-degrading component is a thermostable, metal-independent enzyme, retaining catalytic activity at elevated processing temperatures and functioning without the requirement for exogenous metal ions. These characteristics underscore its strong potential for practical application in mycotoxin detoxification.

### 3.3. Temperature and pH Stability of the S09T2 Cell Lysate

**Figure 5:**
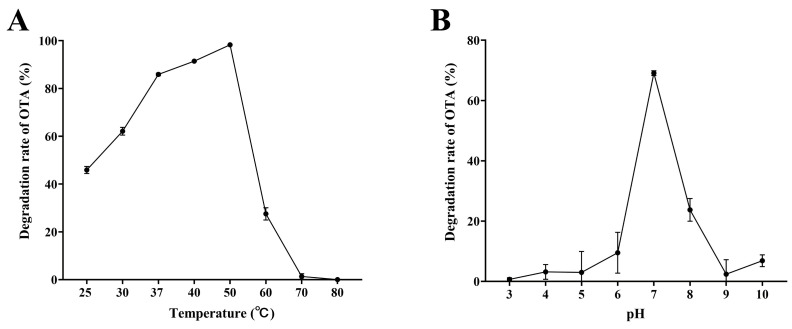
OTA degradation by the cell lysate of S09T2 under different temperature and pH conditions. (**A**) OTA degradation rates after incubation of the S09T2 cell lysate with 1 μg/mL OTA at different temperatures for 6 h. (**B**) OTA degradation rates after incubation of the S09T2 cell lysate with 1 μg/mL OTA at different pH for 6 h at 37 °C. Values are reported as the mean ± standard deviation (SD) (*n* = 3).

To evaluate the thermal stability of the crude enzyme extract from S09T2 strain, its OTA-degrading activity was measured after incubation with 1 μg/mL OTA for 6 h under different thermal conditions (25–80 °C). As shown in Figure 5A, the Enzyme activity increased with temperature, reaching a maximum degradation rate of 98.4% at 50 °C. However, further temperature elevation resulted in a gradual loss of activity, and the cell lysate nearly lost all OTA-degrading capacity at temperatures above 70 °C.

The influence of pH on OTA degradation by the S09T2 lysate was investigated across a range of buffer conditions. As shown in Figure 5B, the lysate exhibited the greatest degradation capability around pH 7.0. Both acidic and alkaline conditions significantly reduced the OTA-degrading efficiency of the cell lysate. Together, these results indicate that the OTA-degrading enzyme in S09T2 cell lysate possesses thermal stability, with an optimal temperature near 50 °C, but exhibits relatively poor pH stability.

### 3.4. Detoxification of OTA-Contaminated Plumeria rubra Extract by S09T2 Cell Lysate

The results of OTA removal by the S09T2 lysate in the *Plumeria rubra* extract system are shown in Supplementary Table S3. In this experiment, equal volumes of OTA-contaminated extract and the bacterial lysate were mixed and incubated at 37 °C for approximately 12 h. The OTA content in the herbal extract was significantly reduced, with a degradation rate of 67.3%. These results demonstrate that the S09T2 cell lysate retains OTA-degrading activity in a complex matrix and shows potential for detoxifying OTA-contaminated herbal products.

## 4. Discussion

Although a variety of OTA-degrading microorganisms have been reported, their practical use remains limited. Fungal strains typically degrade OTA very slowly, often requiring more than 72 h to achieve substantial toxin reduction [37,53]. Bacterial strains generally act faster, with many achieving high degradation levels within approximately 48 h; however, most studies evaluate them only at relatively low OTA concentrations, usually below 2.5 μg/mL [30,34,35]. Moreover, systematic assessments of strain safety (e.g., hemolytic activity, virulence, or resistance genes) have rarely been conducted, which limits their practical applicability [32,36]. Against this backdrop, we report for the first time that *Brevibacillus parabrevis* S09T2 exhibits highly efficient OTA-degrading activity, achieving a 93% reduction of 8 μg/mL OTA within only 24 h of incubation. UPLC-HRMS analysis confirmed that OTA degradation by S09T2 yielded only OTα and phenylalanine, with no other by-products detected (Figure 2 and Figure 3), and this degradation process was attributed to intracellular enzymatic activity (Figure 4). Furthermore, hemolysis testing and whole-genome sequencing revealed no evidence of hemolytic activity, virulence factors, or antimicrobial resistance genes in S09T2, supporting its biosafety. Interestingly, Chucen Liu et al. [33] also isolated two OTA-degrading strains from wheat, both belonging to *Brevibacillus* genus. Therefore, exploring enzymes from this genus could expand the enzymatic resource pool and provide new candidates for OTA detoxification. Members of the *Brevibacillus* genus are additionally recognized as potential biocontrol agents due to their ability to produce diverse bioactive metabolites, conferring them broad application prospects in agriculture, aquaculture, and biotechnology [54]. For example, *Brevibacillus parabrevis* KAU2022 effectively suppressed *Fusarium oxysporum* (FOC7) infection in cumin and enhanced seed germination [55]. Moreover, *B. parabrevis* has been reported to degrade a variety of environmental pollutants, including beta-cypermethrin [56], congo red dye [57], and deltamethrin [58]. Although *B. parabrevis* is generally regarded as a low-risk and safe microorganism, comprehensive toxicological and genomic safety assessments are still necessary to support its potential applications.

Importantly, the investigation of OTA removal should not be limited to controlled laboratory conditions but should also be validated in real food matrices. Two major biological strategies have been proposed for controlling OTA contamination in food products. One strategy relies on antagonistic microorganisms to suppress the growth of OTA-producing fungi, thereby minimizing the risk of contamination. For example, Cubaiu et al. [59] demonstrated that *Saccharomyces cerevisiae* DISAABA1182 effectively suppressed *Aspergillus carbonarius* infection in grapes and significantly reduced OTA production. Similarly, Eamlaor et al. [60] found that volatile organic compounds (VOCs) produced by antagonistic yeasts could inhibit fungal growth and OTA biosynthesis. Their study showed that the application of VOCs on coffee beans and maize kernels led to over 99% reduction in OTA levels. Another strategy involves microbial adsorption or enzymatic degradation to detoxify OTA-contaminated food products. For example, Zheng et al. [61] reported that *Lactobacillus rhamnosus* Bm01 could effectively adsorb OTA via its cell wall components. After 48 h of treatment, nearly 100% of OTA was removed from both grape juice and ready-to-drink coffee. Zou et al. [37] reported that *Aspergillus niger* FS-UV-21, an OTA-degrading strain, was capable of removing 59.8% of OTA from contaminated wheat bran. In addition, Sánchez-Arroyo et al. [62] successfully expressed and purified an OTA-degrading amidohydrolase (AnOTA) from *Aspergillus niger*, which was capable of removing OTA from commercial plant-based beverages without interfering with beverage proteins. In addition to conventional food and feed products, OTA contamination is increasingly detected in functional herbal products, posing potential health risks to consumers [49,63]. However, microbial strategies for OTA detoxification in herbal matrices remain largely underexplored. In this study, a *Plumeria rubra* sample previously identified in our laboratory as naturally contaminated with OTA was used to evaluate the OTA-removal efficiency of the S09T2 strain. As shown in Table S3, the S09T2 cell lysate effectively reduced OTA levels in the *Plumeria rubra* extract, demonstrating its potential for practical application in the detoxification of herbal materials. In practice, this strain could be applied during the cultivation or postharvest handling of herbs—for instance, by spraying S09T2 onto plant surfaces to suppress OTA accumulation. Moreover, prior to processing, herbal materials could be treated with the S09T2 cell lysate, enabling the intracellular OTA-degrading enzyme to remove residual OTA and thereby enhance product safety. Future work will focus on further evaluating the feasibility of these strategies, including assessing whether OTA detoxification can be achieved without altering the levels of bioactive constituents in herbal products.

In summary, we isolated a novel OTA-degrading strain, *Brevibacillus parabrevis* S09T2, from soil, which has not been previously reported. UPLC-HRMS analysis confirmed that this strain employs an intracellular enzyme to degrade OTA into OTα and phenylalanine, without generating any by-products. Notably, S09T2 exhibited high degradation efficiencies across a wide range of OTA concentrations (1–10 µg/mL), achieving nearly 93% degradation within 24 h at 8 µg/mL OTA. Furthermore, the cell lysate of S09T2 effectively eliminated OTA in complex matrices, highlighting its potential for application in real food and feed systems. Future work will focus on protein purification and proteomic approaches to identify potential OTA-degrading enzymes from S09T2, as well as systematic assessments of strain stability and safety to evaluate its suitability for direct application in food systems. These efforts aim to expand both microbial and enzymatic resources for OTA detoxification.

## 5. Conclusions

In this study, *Brevibacillus parabrevis* S09T2 was identified as a previously unreported and highly efficient OTA-degrading bacterium. When the initial OTA concentrations were 5 or 8 μg/mL, the S09T2 fermentation broth achieved over 93% degradation within 24 h. The strain rapidly degraded OTA and generated the far less toxic products OTα and phenylalanine, thereby achieving effective detoxification. OTA removal was mediated by a thermostable intracellular enzyme. In addition, S09T2 exhibited a favorable safety profile, showing no hemolytic activity and carrying no detectable virulence or antimicrobial resistance genes. Notably, the S09T2 lysate effectively reduced OTA levels in naturally contaminated *Plumeria rubra* extract, demonstrating strong potential for practical application in food, feed, and herbal materials. Future work will focus on assessing the strain’s stability and biosafety, characterizing its OTA-degrading enzyme, and further evaluating its detoxification performance in real-world samples.

## Data Availability

The original contributions presented in the study are included in the article/Supplementary Material; further inquiries can be directed to the corresponding authors.

## Supplementary Materials

The following supporting information can be downloaded at: https://www.mdpi.com/article/10.3390/foods15020295/s1, Figure S1: Screening of OTA-degrading strains. (A) Residual OTA levels in each sample after 5 days of incubation in MSM with OTA as the sole carbon source. (B) Single colonies isolated from the most effective sample (S09) were incubated in LB medium containing 1 μg/mL OTA at 37 °C for 3 days, and their OTA degradation efficiencies were evaluated; Table S1: MS/MS parameters for OTA and OTα; Table S2: Validation results of method parameters for the quantification of OTA and OTα; Table S3: Detoxification efficiency of S09T2 cell lysate on OTA-contaminated *Plumeria rubra* extract.

## Abbreviations

The following abbreviations are used in this manuscript:

OTA	Ochratoxin A
OTα	Ochratoxin α
UPLC-MS/MS	Ultra-high-performance liquid chromatography–Tandem Mass Spectrometry
UPLC-HRMS	Ultra-high-performance liquid chromatography–high-resolution mass spectrometry
EDTA	Ethylenediaminetetraacetic acid
