# Supplementary material for: Isolation and Characterization of Brevibacillus parabrevis S09T2, a Novel Ochratoxin A-Degrading Strain with Application Potential
Source: Foods. 2026 Jan 14;15(2):295. doi: 10.3390/foods15020295 (PMC12841047; doi:10.3390/foods15020295)
Supplement: Supplementary file 1 [file foods-15-00295-s001.zip › foods-4007077-supplementary.pdf]

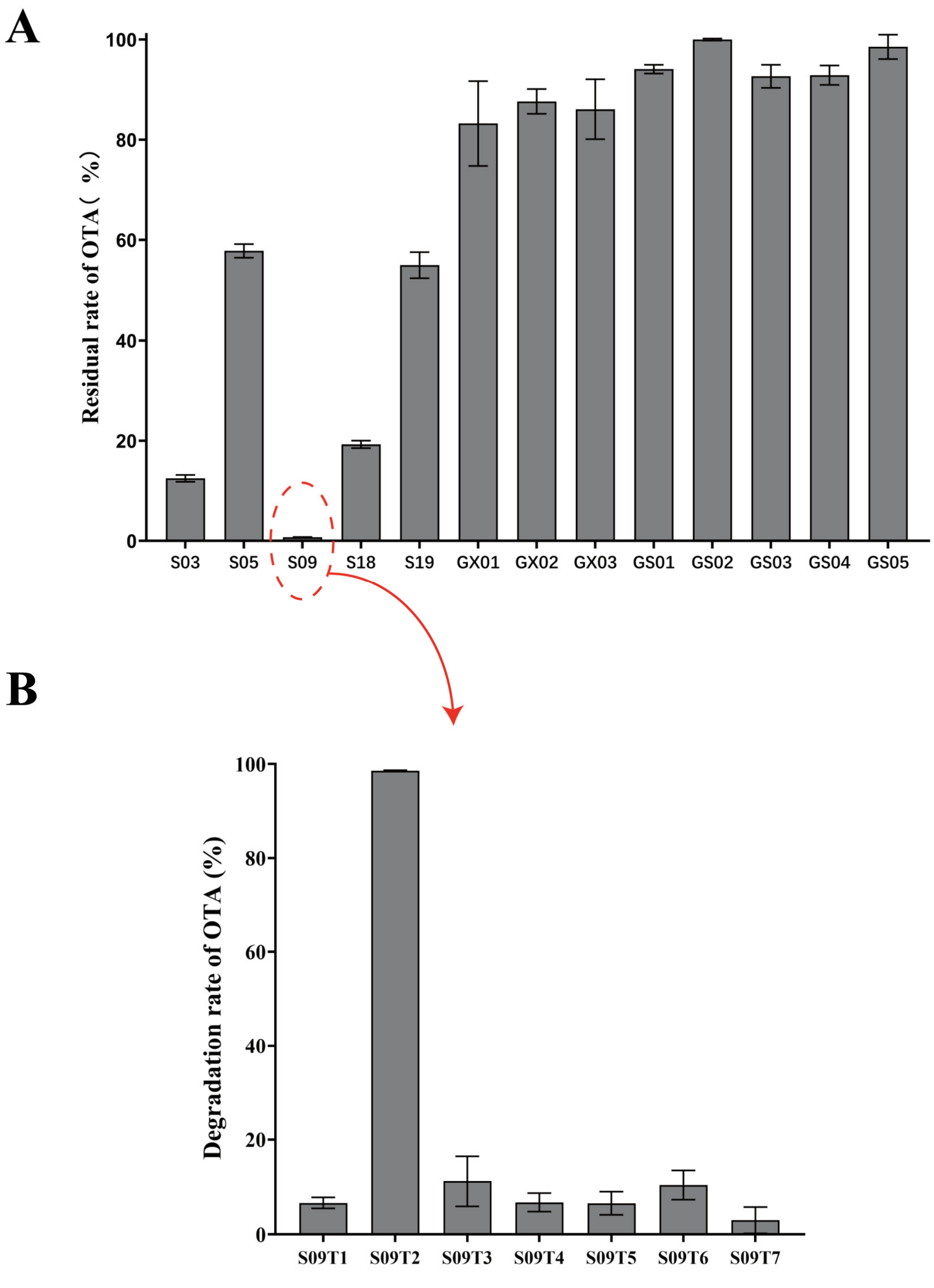

Fig. S1. Screening of OTA-degrading strains. (A) Residual OTA levels in each sample after 5 days of incubation in MSM medium with OTA as the sole carbon source. (B) Single colonies isolated from the most effective sample (S09) were incubated in LB medium containing 1 μg/mL OTA at 37 °C for 3 days, and their OTA degradation efficiencies were evaluated.

Table. S1 MS/MS parameters for OTA and OTα

Analyte	Precursor ion	Fragment ion	DP	CE
OTA	402.1 (-H)	358.1	-60	-29
		211.1	-60	-40
OTα	255 (-H)	211	-60	-22
		167	-60	-34

Note: “DP” declustering potential; “CE” collision energies

Table. S2 Validation results of method parameters for the quantification of OTA and OTα

Analyte	LOD (μg/L)	LOQ (μg/L)	Calibration curves	R^2	Linear range (μg/L)	Concentration (μg/L)	Recovery (%)	RSDr (%)	ME (%)
OTA	0.1	0.5	y = 24437x - 3478.9	0.9997	1-200	10.0	92.91 ± 4.39	6.04	8.19
						100.0	97.77 ± 9.36		
						1000.0	102.28 ± 7.33		
OTα	0.2	0.5	y = 14355x - 3867.5	0.9998	1-200	10.0	93.17 ± 2.81	7.12	11.97
						100.0	100.52 ± 6.48		
						1000.0	98.32 ± 7.47		

Note: "LOD" limit of detection; "LOQ" limit of quantification; "R2" coefficient of determination; "RSDr" relative standard deviation of repeatability; "ME" matrix effect.

Table. S3 Detoxification efficiency of S09T2 cell lysate on OTA-contaminated *Plumeria rubra* extract

Sample	OTA Concentration in Control Group (μg/L)	OTA Concentration in Treatment Group (μg/L)	Degradation Rate (%)
1	0.928	0.239	74.3
2	0.698	0.239	65.8
3	0.785	0.301	61.7
Average ± SD	0.804 ± 0.116	0.260 ± 0.036	67.2 ± 6.4

Note: 1 mL of *Plumeria rubra* extract was incubated with 1 mL of PBS (control) or 1 mL of S09T2 cell lysate (treatment) at 37 °C overnight, followed by OTA quantification.
